# Screening of Potential Thrombin and Factor Xa Inhibitors from the Danshen–Chuanxiong Herbal Pair through a Spectrum–Effect Relationship Analysis

**DOI:** 10.3390/molecules26237293

**Published:** 2021-12-01

**Authors:** Xu Wang, Dai-Yan Zhang, Shi-Jun Yin, Hui Jiang, Min Lu, Feng-Qing Yang, Yuan-Jia Hu

**Affiliations:** 1 School of Chemistry and Chemical Engineering, Chongqing University, Chongqing 401331, China; 201818021128@cqu.edu.cn (X.W.); 201718021124@cqu.edu.cn (S.-J.Y.); 20181802008t@cqu.edu.cn (H.J.); 201818021134@cqu.edu.cn (M.L.); 2 State Key Laboratory of Quality Research in Chinese Medicine, Institute of Chinese Medical Sciences, University of Macau, Macao SAR 999078, China; yc07528@umac.mo

**Keywords:** Danshen–Chuanxiong herbal pair, thrombin inhibitors, factor Xa inhibitors, spectrum–effect relationship analysis

## Abstract

In this study; a spectrum–effect relationship analysis combined with a high-performance liquid chromatography–mass spectrometry (LC–MS) analysis was established to screen and identify active components that can inhibit thrombin and factor Xa (THR and FXa) in Salviae Miltiorrhizae Radix et Rhizoma–Chuanxiong Rhizoma (Danshen–Chuanxiong) herbal pair. Ten potential active compounds were predicted through a canonical correlation analysis (CCA), and eight of them were tentatively identified through an LC–MS analysis. Furthermore; the enzyme inhibitory activity of six available compounds; chlorogenic acid; *Z*-ligustilide; caffeic acid; ferulic acid; tanshinone I and tanshinone IIA; were tested to verify the feasibility of the method. Among them; chlorogenic acid was validated to possess a good THR inhibitory activity with IC_50_ of 185.08 µM. Tanshinone I and tanshinone IIA are potential FXa inhibitors with IC_50_ of 112.59 µM and 138.19 µM; respectively. Meanwhile; molecular docking results show that tanshinone I and tanshinone IIA; which both have binding energies of less than −7.0 kcal·mol^−1^; can interact with FXa by forming H-bonds with residues of SER214; GLY219 and GLN192. In short; the THR and FXa inhibitors in the Danshen–Chuanxiong herbal pair have been successfully characterized through a spectrum–effect relationship analysis and an LC–MS analysis.

## 1. Introduction

Thrombin (THR) and factor Xa (FXa), which are members of the Serine Protease family, play critical roles in the coagulation cascade [1]. In thrombotic diseases, THR catalyzes the transformation of soluble fibrinogen into insoluble fibrin in response to a vascular injury, leading to the formation of a clot [2]. FXa is located at the center of endogenous and exogenous coagulation pathways. It is worth mentioning that the formation of THR can also be reduced by inhibiting its activity to prevent the expansion of a thrombus [3]. In reality, thrombotic disease, a common type of cardiovascular disease, has gradually become a threat to public health [4]. Due to the importance of THR and FXa in the formation of thrombosis, inhibiting their activity is a feasible strategy for the treatment of thrombotic diseases [5]. At present, anticoagulant drugs including argatroban (THR inhibitor) and rivaroxaban (FXa inhibitor) are used clinically [6,7], but risks such as drug resistance and gastrointestinal bleeding may be caused by these inhibitors [8]. Therefore, it has become a trend to search for THR and FXa inhibitors in natural sources to provide a reference for the development of anticoagulant drugs [9].

Danshen (Salviae Miltiorrhizae Radix et Rhizoma, DS) and Chuanxiong (Chuanxiong Rhizoma, CX) are both effective for invigorating blood circulation and eliminating blood stasis [10]. DS and CX have attracted widespread attention as primary materials for the treatment of cardiovascular diseases (CVDs) [11], tumor disorders [12] and inflammatory diseases [13]. DS is often employed clinically combined with other traditional Chinese medicines (TCMs) for promoting blood circulation, of which Danshen–Chuanxiong (DC) is one of the most frequently used herbal pairs (mixture of plant materials) among best-selling herbal formulae [14]. In our previous studies, potential THR and FXa inhibitors in DS and CX were screened, respectively [1,5]. However, the THR and FXa inhibitory activity of the DC herbal pair has not been evaluated, and the potential active components are still unclear. Therefore, a spectrum–effect relationship analysis was performed to screen the active ingredients in the DC herbal pair that inhibit THR and FXa. A spectrum–effect relationship analysis [15], correlating the peak area in the fingerprint of TCMs with pharmacodynamic data, is employed to reveal the correlation between the components and their pharmacological activities. Due to the advantages of being a simple operation and saving time [16], spectrum–effect relationship analyses have been reliably used in the screening of natural active compounds. For instance, protocatechuic aldehyde, hydroxysafflor yellow A and tanshinone IIA were screened and identified as active tyrosinase inhibitors in the *Salvia miltiorrhiza*–*Carthamus tinctorius* herbal pair through a spectrum–effect relationship analysis [17].

In this study, the inhibition effects of the DC herbal pair with different ratios on THR and FXa were compared. Then, the components in the DC herbal pair were analyzed through HPLC and the chemical fingerprint was constructed. A spectrum–effect relationship analysis was employed to predict the potential active compounds in the DC herbal pair, and their chemical structures were identified through an LC–MS analysis. Furthermore, the in vitro THR and FXa inhibitory activities of the predicted compounds were tested. Finally, molecular docking was used to explore the possible binding sites and modes of action between the active compounds and THR and FXa, respectively.

## 2. Results and Discussion

### 2.1. Inhibitory Effects of DC Extracts on THR and FXa

**Figure 1:**
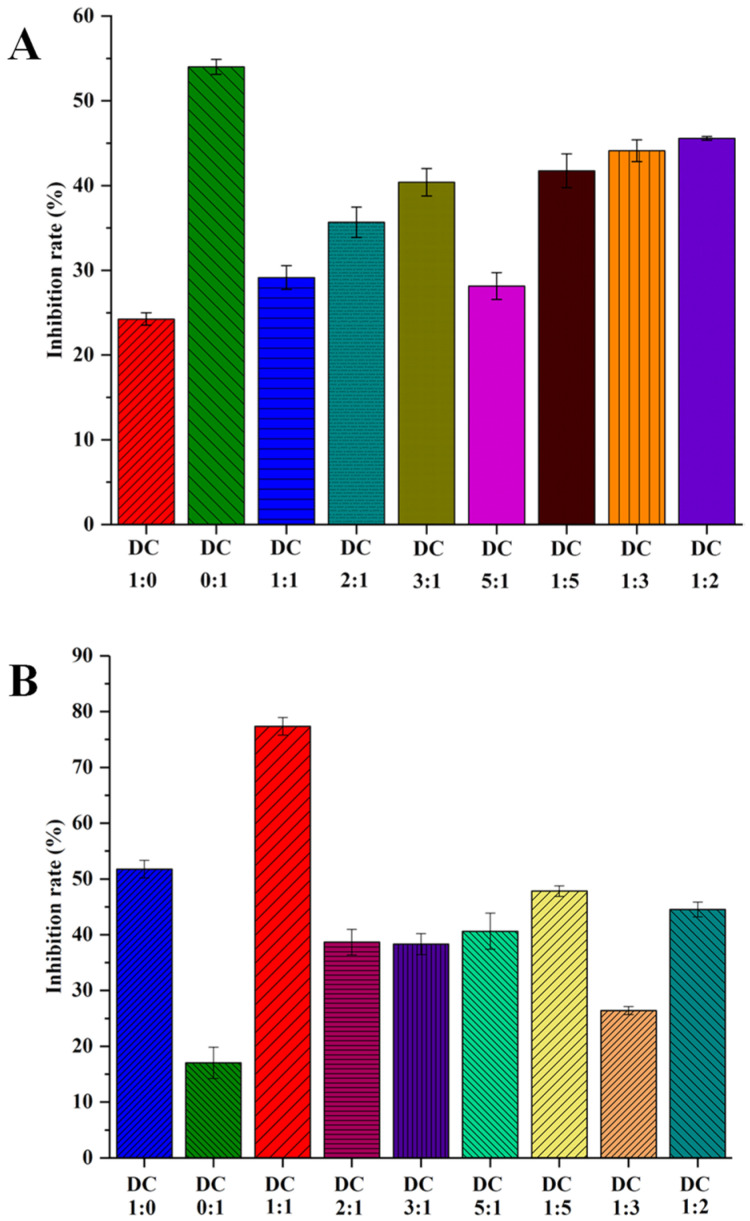
The % inhibition from DC extracts with different proportions of Danshen and Chuanxiong on thrombin (**A**) and factor Xa (**B**).

As shown in Figure 1A, different ratios of DC herbal pair extracts show a weaker inhibitory effect on THR than the CX extract when the final concentration is 2.5 mg/mL. However, compared with the DS extract, all ratios of DC herbal pair extracts display stronger inhibitory activity on THR. The DC herbal pair extracts (1:1 and 5:1) display a weaker effect than the other DC herbal pair extracts (2:1, 3:1, 1:5, 1:3 and 1:2). As shown in Figure 1B, different ratios of DC herbal pair extracts show a stronger inhibitory effect on FXa than the CX extract when the final concentration is 2.5 mg/mL. Among them, the DC herbal pair (1:1) exhibits the strongest effect, even stronger than the DS extract. However, the inhibitory effects of other DC herbal pair extracts (2:1, 3:1, 5:1, 1:5, 1:3 and 1:2) on FXa are weaker than the DS extract. In reality, studies show that the combined use of DS and CX display a better antithrombotic effect at a ratio of 1:1 than their separate application [14], indicating the mechanism for the DC herbal pair to exhibit an antithrombotic effect may be by inhibiting the activity of FXa. The results may provide scientific evidence for the experience-based application of the DC herbal pair [18]. 

### 2.2. Spectrum–Effect Relationship Analysis

**Figure 2:**
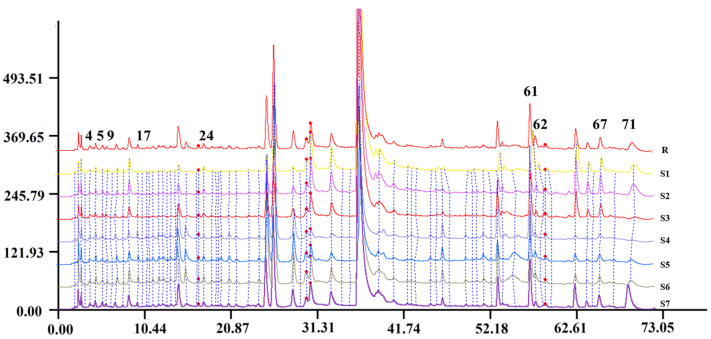
Aligned HPLC chromatograms of DC extracts with seven proportions of Danshen and Chuanxiong. The chromatograms of S1–S7 are as follows: DC 1:1 (S1); DC 1:2 (S2); DC 1:3 (S3); DC 1:5 (S4); DC 2:1 (S5); DC 3:1 (S6); DC 5:1 (S7); and control map (R).

The HPLC fingerprints of DC samples with different ratios are shown in Figure 2. A canonical correlation analysis (CCA) was used to assess the spectrum–effect relationship between the areas of 71 chromatographic peaks and the inhibition rate of different extracts on THR and FXa. As shown in Table 1, the results are expressed through a Pearson correlation coefficient; “r” indicates the correlation strength, 0.6 ≤ |r| ≤ 0.8 indicates a strong correlation and 0.8 ≤ |r| ≤ 1.0 indicates a significant correlation [19]. Therefore, compounds of peaks 4, 17, 24, 55, 61 and 62 with ”r” higher than 0.8 may be the active components of herbal pairs for inhibiting THR. On the other hand, compounds of peaks 5, 9, 61, 67 and 71 with ”r” higher than 0.6 are potential active FXa inhibitory compounds. A further LC–MS analysis was performed to identify the structures of these compounds, and the inhibitory activity of the pure compounds was tested in vitro.

### 2.3. Identifications of the Potential Active Compounds through an LC–MS Analysis

**Figure 3:**
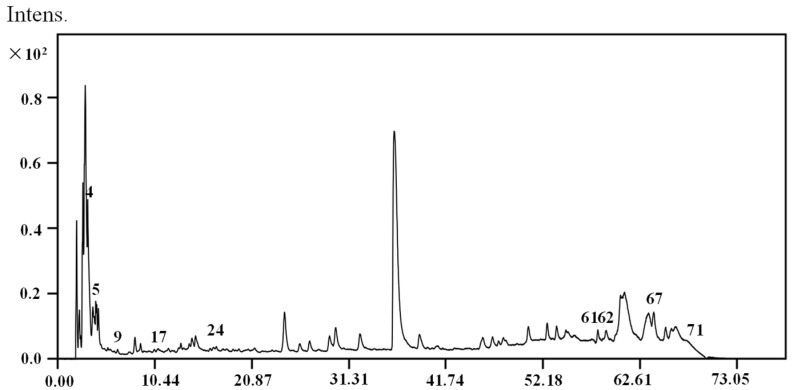
Total ion chromatograms of the DC (1:2) extract.

**Figure 4:**
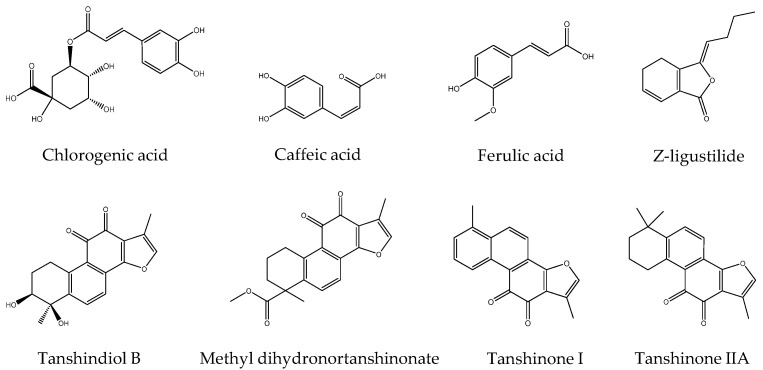
Chemical structures of the predicted compounds in DC herbal pair.

An LC–MS analysis was used to study the chemical structures of potential active compounds; the total ion chromatogram was shown in Figure 3. Eight compounds, chlorogenic acid, caffeic acid, ferulic acid, *Z*-ligustilide, tanshindiol B, methyl dihydronortanshinonate, tanshinone I and tanshinone IIA, were tentatively identified by comparing their MS data with those reported in the literature [20,21,22]. The detailed MS data of ten predicted compounds are shown in Table 2, and their chemical structures are presented in Figure 4.

Peak 4 gives a [M − H]^−^ ion at *m/z* 353.18; the product ion is obtained at *m/z* 191.06 for [M − H − C_9_H_6_O_3_]^−^, at *m/z* 179.06 for [M − H − C_7_H_10_O_5_]^−^ and at *m/z* 135.03 for [M–H − C_8_H_10_O_7_]^−^. Therefore, peak 4 is suspected to be chlorogenic acid [20]. Peak 5 has [M − H]^−^ at *m/z* 179.06; the product ion is obtained at *m/z* 135.03 for [M − H − C_2_H_4_O]^−^. Peak 5 is tentatively identified as caffeic acid [21]. Peak 9 shows a [M − H]^−^ ion at *m/z* 193.05, which yields an MS^2^ fragment ion at *m/z* 149.01 for [M + H − HCOOH]^−^. According to the reference [20], peak 9 is suspected to be ferulic acid. Peak 17 yields a [M − H]^−^ ion at *m/z* 191.07; the product ion is obtained at *m/z* 172.02 for [M + H − H_2_O]^−^. Therefore, peak 17 is suspected to be *Z*-ligustilide [20]. Peak 61 shows a peak of [M − H]^−^ ions at *m/z* 311.24; the product ion is obtained at *m/z* 265.16 for [M − H − C_2_H_6_O]^−^. Therefore, peak 61 is suspected to be tanshindiol B [21]. Peak 62 displays a [M − H]^−^ ion at *m/z* 339.22; the product ion is obtained at *m/z* 261.13 for [M + H − C_3_H_11_O_2_]^−^. Therefore, peak 62 is tentatively identified as methyl dihydronortanshinonate [21]. Peak 67 displays a [M − H]^−^ ion at *m/z* 277.2; the product ion is obtained at *m/z* 248.99 for [M − H − CO]^−^. Therefore, peak 67 is suspected to be tanshinone I [22]. Peak 71 shows a [M − H]^−^ ion at *m/z* 293.23, which yields MS^2^ fragment ions at *m/z* 277.23 for [M + H − H_2_O]^−^ and at *m/z* 248.99 for [M + H − H_2_O − CO]^−^. Therefore, peak 71 is suspected to be tanshinone IIA [22].

### 2.4. In Vitro Activity Tests for the Predicted Compounds

**Figure 5:**
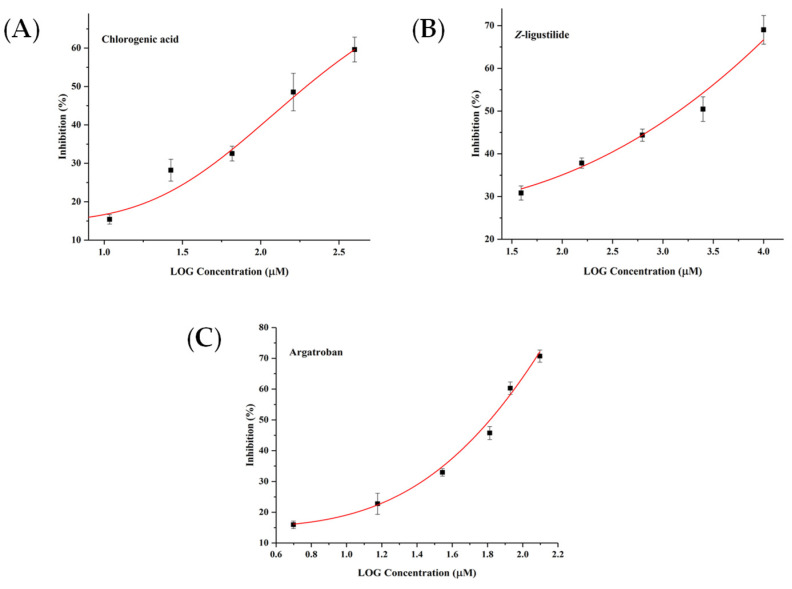
The inhibitory activity of chlorogenic acid (**A**), Z-ligustilide (**B**) and argatroban (**C**) on thrombin.

**Figure 6:**
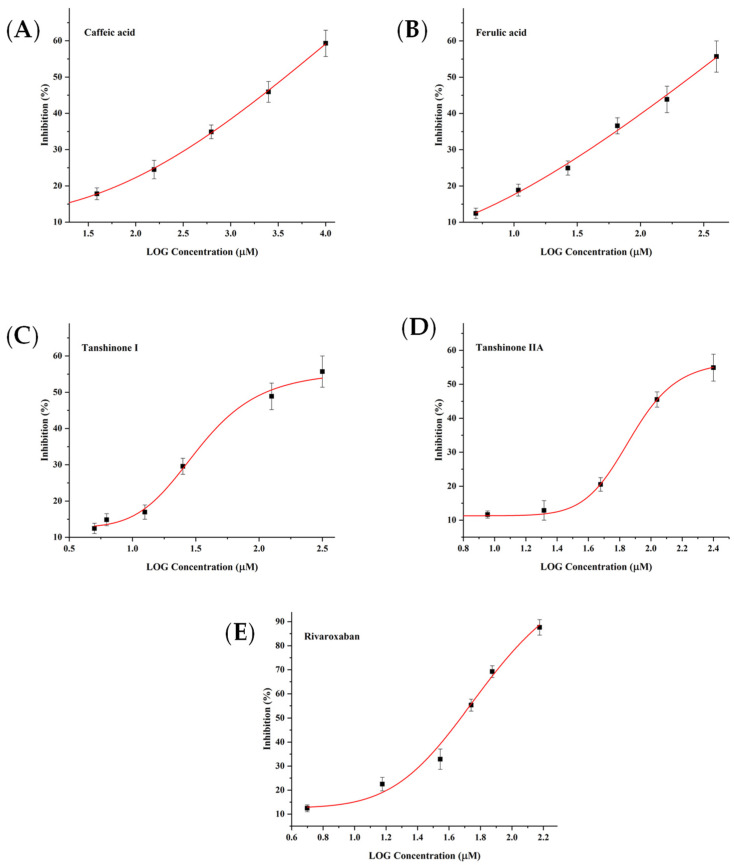
The inhibitory activity of caffeic acid (**A**), ferulic acid (**B**), tanshinone I (**C**), tanshinone IIA (**D**) and rivaroxaban (**E**) on FXa.

In order to confirm the feasibility of the spectrum–effect relationship method for screening of active compounds, in vitro enzymatic activity assays were performed. Argatroban, a well-known THR inhibitor [6], was selected as the positive control drug. The results are shown in Figure 5. Argatroban shows a strong inhibition effect with an IC_50_ value of 64.94 µM. Chlorogenic acid with an IC_50_ value of 185.08 µM exhibits a better inhibitory activity than *Z*-ligustilide with an IC_50_ value of 1460.65 µM on THR. As shown in Figure 6, the IC_50_ value of rivaroxaban, as the positive control drug [7], is 47.21 µM. Among four predicted compounds, tanshinone I and tanshinone IIA strongly inhibit the activity of FXa in a dose-dependent manner with IC_50_ values of 112.59 µM and 138.19 µM, respectively. In addition, caffeic acid and ferulic acid do not show a significant inhibitory effect with IC_50_ values of 3789.96 µM and 2488.90 µM, indicating that they may not act directly on the active center of FXa.

### 2.5. Molecular Docking of THR and FXa and Identified Active Compounds

**Figure 7:**
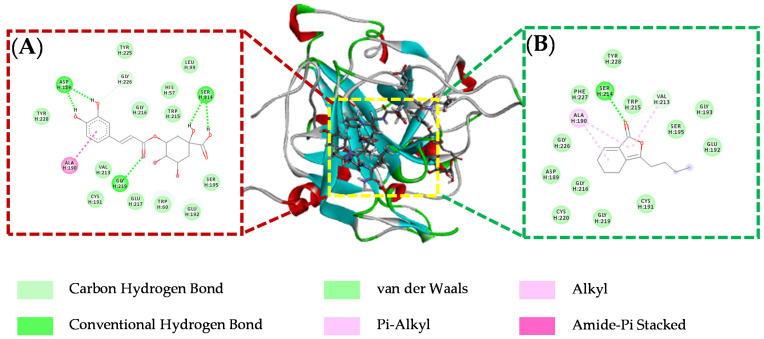
Binding modes and interactions between chlorogenic acid (**A**), Z-ligustilide (**B**) and the thrombin catalytic site.

**Figure 8:**
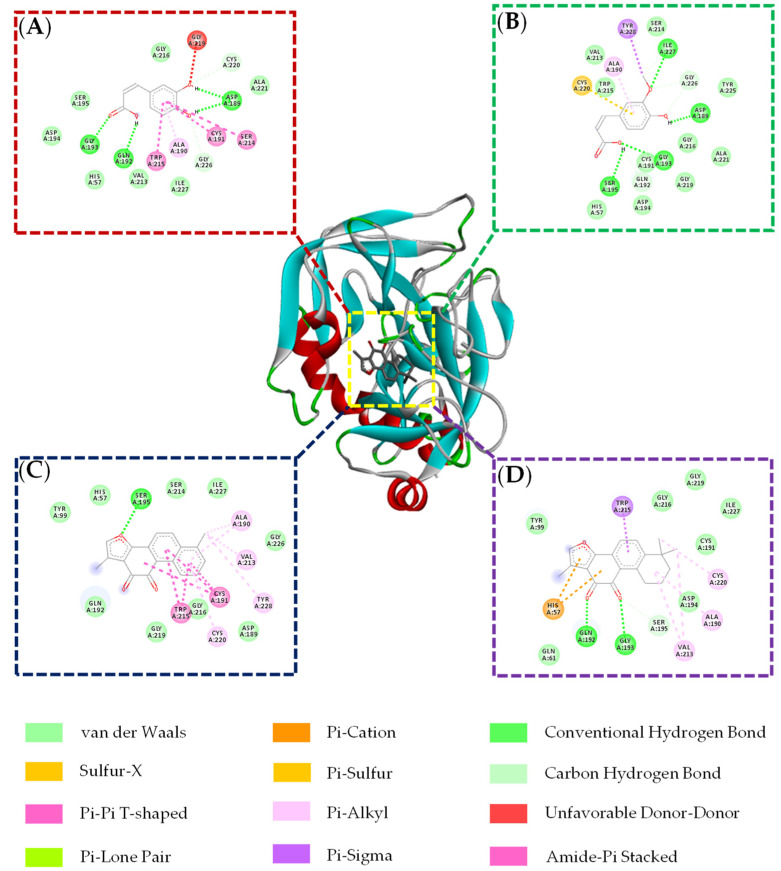
Binding modes and interactions between caffeic acid (**A**), ferulic acid (**B**), tanshinone I (**C**), tanshinone IIA (**D**) and the factor Xa catalytic site.

Molecular docking can be used to reveal the possible modes and sites of compounds interacting with THR and FXa. As shown in Table 3, the binding energies of chlorogenic acid and *Z*-ligustilide are less than −5.0 kcal·mol^−1^, indicating that they are potential THR inhibitors. Chlorogenic acid is combined with THR through Van der Waals forces and ionic bonds. *Z*-ligustilide can enter THR catalytic activity pockets with a strong affinity by forming H-bonds with residues of SER214 and GLY226. The 2D interaction diagrams of chlorogenic acid and *Z*-ligustilide with THR can be observed in Figure 7. The binding energies and residues of caffeic acid, ferulic acid, tanshinone I and tanshinone IIA with FXa are summarized in Table 4. Among them, tanshinone I and tanshinone IIA, which have binding energies of less than −7.0 kcal·mol^−1^, can interact with FXa by forming H-bonds with residues of SER214, GLY219 and GLN192. In addition, caffeic acid and ferulic acid, which have binding energies of −5.82 kcal·mol^−1^ and −6.35 kcal·mol^−1^, are combined with FXa through Van der Waals and other forces. It is worth mentioning that the molecular docking results of the four active components are consistent with their activity in in vitro tests. Tanshinone I and tanshinone IIA with stronger affinity exhibit better inhibition effects. The 2D interaction diagrams are shown in Figure 8.

## 3. Materials and Methods

### 3.1. Chemicals and Materials

Thrombin was purchased from Sigma-Aldrich (St. Louis, MO, USA). Substrate S-2238 (≥99%, determined through HPLC) was bought from Adhoc International Technology Co., Ltd. (Beijing, China). Factor Xa and substrate S-2765 (≥99%, determined through HPLC) were obtained from Adhoc International Technology Co., Ltd. (Beijing, China). Argatroban was bought from Huazhong Haiwei Gene Technology Co., Ltd. (Beijing, China). Rivaroxaban was from Aladdin Biochemical Technology Co., Ltd. (Shanghai, China). The reference compounds of chlorogenic acid, *Z*-ligustilide, caffeic acid, ferulic acid, tanshinone I and tanshinone IIA (≥98%, determined through HPLC) were purchased from Purechem-standard Co., Ltd. (Chengdu, China). HPLC-grade acetonitrile was purchased from Shanghai Titan Scientific Co., Ltd. (Shanghai, China). Water used for all the experiments was purified by a water purification system (ATSelem 1820A, Antesheng Environmental Protection Equipment, Chongqing, China). All the other chemicals and solvents, such as ethanol, potassium dihydrogen phosphate and disodium phosphate, were obtained from Chron Chemicals Co., Ltd. (Chengdu, China).

Crude drugs (pieces of plant materials) of Danshen and Chuanxiong were purchased from Kangmei Pharmaceutical Co., Ltd. in May 2020. Their origins are from Shandong and Sichuan, respectively. The voucher specimens of *Salvia miltiorrhiza* Bge. (number SM2020050101) and *Ligusticum chuanxiong* Hort. (number CF2020050101) were deposited at the Pharmaceutical Engineering Laboratory in School of Chemistry and Chemical Engineering, Chongqing University, Chongqing, China.

### 3.2. Preparation of DC Extracts 

Crude drugs of DS and CX were pulverized, then stored in a dry box after filtrating through a 50 mesh sieve (about 0.29 mm). Nine different ratios of DC herbal pairs (1:0, 1:1, 2:1, 3:1, 5:1, 1:5, 1:3, 1:2 and 0:1) were prepared for reflux extraction. First, 2.5 g of DS and CX mixed powder was extracted through ultrasonic extraction at room temperature for 15 min with 20 mL of 70% ethanol in a 250 mL round-bottom flask. Then, it was further extracted in a water bath at 80 °C for 30 min. The above process was repeated three times. After the extract solutions were filtered through filter paper and combined, they were evaporated in a rotary evaporator (ZFQ 85A, Shanghai Medical Instrument Special Factory, Shanghai, China) at 55 °C under reducing pressure to remove the ethanol solvent. Finally, the extracts were further dried by lyophilization with a freezing–drying system (DZF-6050, Shanghai Jing Hong Laboratory Instrument Co., Ltd., Shanghai, China) to obtain the DC extracts at a yield of about 20% (*w*/*w*, dried extract/crude herb). All solutions were stored in the dark at 4 °C. Before the HPLC analysis, the extract was dissolved in methanol (5.0 mg/mL) and filtered through a 0.22 μm membrane filter (Shanghai Titan Scientific Co., Ltd., Shanghai, China).

### 3.3. HPLC and LC–MS Analysis

An HPLC analysis was performed on an Agilent 1260 series liquid chromatograph system (Agilent Technologies, Palo Alto, CA, USA), which is equipped with a diode array detector (DAD), controlled with Agilent ChemStation software. Chromatographic separation was operated on an Agilent ZOBAX SB-Aq column (250 mm × 4.6 mm, 5 μm) preceded by a guard column (12.5 mm × 4.6 mm, 5 μm) at a column temperature of 35 °C. The mobile phase, which consists of 0.1% aqueous acetic acid solution (A) and acetonitrile (B), was at a flow rate of 1.0 mL/min. The gradient program was programmed as follows: 0–2 min, 5% B; 2–10 min, 5%–15% B; 10–24 min, 15%–22% B; 24–35 min, 22%–29% B; 35–42 min, 29%–37% B; 42–60 min, 37%–60% B; 60–62 min, 60%–5% B; and 62–70 min, 5% B. The injection volume for all samples was 10.0 μL.

An Agilent 6545 equipped with electrospray ionization was used for LC/Q-TOF-MS analyses (Santa Clara, CA, USA). The LC conditions were the same as described previously. Nitrogen was used as the atomizing dry gas at a flow rate of 8.0 L/min. The Q-TOF-MS analysis was used in both negative and positive ion modes. The key parameters were set as follows: ion source temperature, 320 °C; sprayer pressure, 35 psi; spray voltage, 1000 V; and TOF acquisition speed, 0.5 s. For compound identification, MS^1^ data and MS^2^ data were recorded in the range of *m/z* 100 to 1000. An LC–MS software package (Agilent) was employed for data collection and processing.

### 3.4. THR and FXa Inhibitory Activity Assay

#### 3.4.1. Preparation of Buffers and Solutions

A PBS buffer was prepared by dissolving 1.44 g of Na_2_HPO_4_ and 0.24 g of KH_2_PO_4_ in 800 mL of water for immediate use (pH was adjusted to 8.0 by 1.0 M NaOH). A stock solution of THR (125 U/mL) was prepared by dissolving 4.16 mg of THR in 1.0 mL of PBS. A stock solution of FXa (0.5 IU/mL) was prepared by dissolving 5.0 mg of FXa in 1.0 mL of PBS. Stock solutions of S-2238 (2 mg/mL) and S-2765 (2.5 mg/mL) were prepared in PBS. The DC extract samples (10 mg/mL) were prepared in a PBS buffer and diluted to 5.0 and 2.5 mg/mL as necessary.

#### 3.4.2. Calculation of the % Inhibition

THR and FXa inhibitory activity assays were performed with an iMark™ Microplate Absorbance Reader (Bio-Rad Laboratories, Inc., Hercules, CA, USA). A DC extract solution of 50 μL and a 50 μL THR solution were mixed and incubated for 10 min at 37 °C. Subsequently, a 100 μL S-2238 (2 mg/mL) solution as the chromogenic substrate was added to start the reaction in the microplate reader. The absorbance was monitored at 405 nm every 30 s for 10 min, and each group was measured three times. As a control group, the operation process was similar to the experimental group but without a DC extract. Except for the enzyme solution and chromogenic substrate, the procedure for assessing the FXa inhibitory activity was exactly the same as for THR. The inhibition rate (%) of THR and FXa was calculated using Formula (1).

(1) $$I\left(\%\right)=\frac{\left[{\left(\frac{dA}{dt}\right)}_{blank}-{\left(\frac{dA}{dt}\right)}_{sample}\right]}{{\left(\frac{dA}{dt}\right)}_{blank}}$$

where I(%) represents the % of inhibition, and ${\left(\frac{dA}{dt}\right)}_{blank}$ and ${\left(\frac{dA}{dt}\right)}_{sample}$ represent the reaction rate of the control and experimental group, respectively. The results were expressed as the mean ± standard deviation (SD) of three experiments.

### 3.5. Spectrum–Effect Relationship Analysis

A spectrum–effect relationship analysis was performed by transferring the DC fingerprint peak area and inhibition rate of THR and FXa into SPSS software (Ver. 24.0, Chicago, IL, USA) for a canonical correlation analysis (CCA). The chromatograms of nine ratios of DC samples were calculated and generated by “Similarity Evaluation System for Chromatographic Fingerprint of Traditional Chinese Medicine” composed by Chinese Pharmacopoeia Committee (Version 2004A). A CCA was used to establish the spectrum–effect relationships between the areas of 71 peaks in the fingerprint and inhibition rate of THR and FXa, respectively, to screen potential active components.

### 3.6. Molecular Docking of THR and FXa and Identified Active Compounds

AutoDock 4.2 (The Scrips Research Institute, La Jolla, CA, USA) was employed for molecular docking studies to predict the site and mode of action of active compounds from THR and FXa, respectively. The brief steps for molecular docking are as follows. First, the crystal structures of THR (No. 1DWC) and FXa (No. 2W26) from the PDB library were downloaded, which were output in PDBQT format after the following operations: the water molecules and the ligands of argatrobana and rivaroxaban were deleted while polar hydrogen was added and the point charge was calculated. Second, the 3D chemical structures of active compounds were drawn by ChemDraw 3D (Ver. 15.0) to minimize energy to prepare ligands. Semi-flexible docking was used to simulate the docking process. The grid size was set to (*x, y, z*) = (60, 60, 60), and the grid centers of THR and FXa were set to (*x, y, z*) = (35.887, 19.178, 18.856) and (*x, y, z*) = (7.951, 5.850, 21.876), respectively. The Lamarckian genetic algorithm (LGA) was employed and the number of GA runs was equal to 50 for finding the most favorable ligand binding orientations. Finally, Discovery Studio 4.5 (BIOVIA, San Diego, CA, USA) was used to observe the interaction of active compounds with THR and FXa.

### 3.7. Statistical Analysis 

All data are presented as the mean ± standard deviations (SD) of at least three different experiments. The statistical analysis was performed by SPSS.

## 4. Conclusions

In this study, the potential THR and FXa inhibitory components in the DC herbal pair were screened by a spectrum–effect relationship analysis. Combined with a canonical correlation analysis, in vitro THR and FXa inhibitory activity test and LC–MS analysis, two potential THR inhibitory active compounds (chlorogenic acid and *Z*-ligustilide) and four potential THR inhibitory active compounds (caffeic acid, ferulic acid, tanshinone I and tanshinone IIA) were predicted. Furthermore, chlorogenic acid, tanshinone I and tanshinone IIA were verified to possess good THR and FXa inhibitory activity with IC_50_ values of 185.08 µM, 112.59 µM and 138.19 µM, respectively. In addition, the molecular docking results, which are consistent with their in vitro activity verification, show that tanshinone I and tanshinone IIA can interact with FXa by forming H-bonds with residues of SER214, GLY219 and GLN192. 

## Figures and Tables

**Table 1:** Correlation coefficients between the chromatographic peak area and % inhibition of thrombin and factor Xa.

**Peak**	1	2	3	4	5	6	7	8	9	10	11	12	13	14	15
**THR**	−0.598	0.727 *	0.715 *	0.831 **	−0.453	0.723 *	0.709 *	−0.734 *	−0.442	0.761 *	−0.356	−0.338	0.580	0.578	0.695 *
**FXa**	−0.212	−0.127	0.132	−0.497	0.628 *	0.175	0.278	0.359	0.750 *	−0.112	0.149	0.093	0.205	−0.566	−0.242
**Peak**	16	17	18	19	20	21	22	23	24	25	26	27	28	29	30
**THR**	0.769 *	0.826 **	0.750 *	0.449	0.679 *	0.758 *	0.743 *	0.645 *	0.812 **	0.747 *	0.690 *	0.756 *	0.779 *	0.694 *	0.574
**FXa**	−0.245	−0.195	−0.394	−0.265	−0.294	−0.264	−0.126	−0.310	−0.142	−0.111	−0.082	−0.292	−0.226	−0.119	0.218
**Peak**	31	32	33	34	35	36	37	38	39	40	41	42	43	44	45
**THR**	−0.484	0.749 *	0.697 *	0.712 *	0.685 *	0.346	0.766 *	−0.570	−0.549	−0.400	0.455	−0.563	−0.420	−0.515	−0.375
**FXa**	0.173	−0.219	−0.119	−0.141	−0.102	−0.326	−0.346	0.092	−0.030	−0.289	−0.083	0.078	0.231	0.104	0.018
**Peak**	46	47	48	49	50	51	52	53	54	55	56	57	58	59	60
**THR**	0.520	0.410	0.723 *	−0.560	0.724 *	0.460	−0.511	0.701 *	0.635 *	0.877 **	0.793 *	0.722 *	0.343	−0.326	0.301
**FXa**	0.077	−0.182	−0.127	0.131	0.181	0.106	0.455	−0.122	0.051	−0.369	−0.309	−0.284	0.300	0.169	0.249
**Peak**	61	62	63	64	65	66	67	68	69	70	71				
**THR**	0.862 **	0.912 *	0.482	−0.170	−0.550	−0.500	−0.781 *	−0.463	0.265	0.422	−0.597				
**FXa**	−0.626 *	−0.310	0.213	0.364	0.138	0.045	0.867 **	0.156	0.410	−0.016	0.703 *				

Note: A Pearson correlation was used. “r” represents the strength; *, 0.6 ≤ |r| ≤ 0.8 means a strong correlation; ** 0.8 ≤ |r| ≤ 1 means a significant correlation.

**Table 2:** LC–MS and MS data of ten predicted compounds from the DC herbal pair.

Peak No.	t_R_ (min)	MW	MS^1^ (*m/z*)	MS^2^ (*m/z*)	Formula	Identification
4	3.599	354	353.18	191.06;179.06;135.03	C_16_H_18_O_9_	Chlorogenic acid
5	4.178	180	179.06	135.03	C_9_H_8_O_4_	Caffeic acid
9	6.768	194	193.05	149.01	C_10_H_10_O_4_	Ferulic acid
17	11.698	192	191.07	173.02	C_12_H_14_O_2_	*Z*-ligustilide
24	17.217	382	381.03	191.07	-	Unknown
55	50.687	-	-	-	-	Unknown
61	57.766	312	311.24	265.16	C_18_H_16_O_5_	Tanshindiol B
62	58.527	340	339.22	261.13	C_20_H_20_O_5_	Methyl dihydronortanshinonate
67	64.584	278	277.23	248.99	C_18_H_12_O_3_	Tanshinone I
71	69.298	294	293.23	277.23; 248.99	C_19_H_18_O_3_	Tanshinone IIA

**Table 3:** Docking results of chlorogenic acid and Z-ligustilide with thrombin.

Compounds	Binding Energy (kcal/mol)	Hydrogen Bond	Van der Waals Forces	Electrostatic Interaction and Other Forces
Chlorogenic acid	−5.81	-	GLY219, CYS191, ASP194, HIS57, GLU192, TRP215	ALA190, CYS220, SER214, LYS224
*Z*-ligustilide	−6.44	SER214, GLY226	GLU192, ASP194, VAL213, TYR228, SER214, CYS220	LYS224, ARG221A, ASP189

**Table 4:** Docking results of caffeic acid, ferulic acid, tanshinone I and tanshinone IIA with FXa.

Compounds	Binding Energy (kcal/mol)	Hydrogen Bond	Van der Waals Forces	Electrostatic Interaction and Other Forces
Caffeic acid	−5.82	-	SER214, ASP194, ILE227, GLY216, ASP189	VAL213, TRP215, CYS191, CYS220
Ferulic acid	−6.35	-	ILE227, GLY226, GLY216, GLY219, TYR99, ILE227,	ALA190, VAL213, TYR228, HIS57, TRP215
Tanshinone I	−7.87	SER214, GLY219	HIS57, SER214, GLN192, TYR99, GLY216, GLY219	CYS191, CYS220, TRP215, ALA190, VAL213
Tanshinone IIA	−7.44	GLY219, GLN192	LYS96, PHE174, THR98, MET180	TRP215, TYR99, GLU97

## Data Availability

The data presented in this study are contained within the article.

## Sample Availability

Samples of the compounds are available from the authors.

## References

[1] Yang Y.Y., Wu Z.Y., Xia F.B., Zhang H., Wang X., Gao J.L., Yang F.Q., Wan J.B. (2020). Characterization of thrombin/factor Xa inhibitors in Rhizoma Chuanxiong through UPLC-MS-based multivariate statistical analysis. Chin. Med..

[2] Liu L., Ma H.Y., Yang N.Y., Tang Y.P., Guo J.M., Tao W.W., Duan J.A. (2010). A series of natural flavonoids as thrombin inhibitors: Structure-activity relationships. Thromb. Res..

[3] Leadley R.J. (2001). Coagulation factor Xa inhibition: Biological background and rationale. Curr. Top Med. Chem..

[4] Gong P.Y., Guo Y.J., Tian Y.S., Gu L.F., Qi J., Yu B.Y. (2021). Reverse tracing anti-thrombotic active ingredients from dried Rehmannia Radix based on multidimensional spectrum-effect relationship analysis of steaming and drying for nine cycles. J. Ethnopharmacol..

[5] Yang Y.Y., Wu Z.Y., Zhang H., Yin S.J., Xia F.B., Zhang Q., Wan J.B., Gao J.L., Yang F.Q. (2020). LC-MS-based multivariate statistical analysis for the screening of potential thrombin/factor Xa inhibitors from Radix Salvia Miltiorrhiza. Chin Med..

[6] Liss D.B., Mullins M.E. (2021). Antithrombotic and antiplatelet drug toxicity. Crit. Care Clin..

[7] Ding Y., Li X., Zhou M., Cai L., Tang H., Xie T., Shi Z., Fu W. (2021). Factor Xa inhibitor rivaroxaban suppresses experimental abdominal aortic aneurysm progression via attenuating aortic inflammation. Vascul. Pharmacol..

[8] Fawaz B., Candelario N.M., Rochet N., Tran C., Brau C. (2016). Warfarin-induced skin necrosis following heparin-induced thrombocytopenia. Baylor Univ. Med. Center Proc..

[9] Wu Z.Y., Zhang H., Yang Y.Y., Yang F.Q. (2020). An online dual-enzyme co-immobilized microreactor based on capillary electrophoresis for enzyme kinetics assays and screening of dual-target inhibitors against thrombin and factor Xa. J. Chromatogr. A..

[10] Chen C., Wang F.Q., Xiao W., Xia Z.N., Hu G., Wan J.B., Yang F.Q. (2017). Effect on platelet aggregation activity: Extracts from 31 Traditional Chinese Medicines with the property of activating blood and resolving stasis. J. Tradit. Chin. Med..

[11] Li Z.M., Xu S.W., Liu P.Q. (2018). Salvia miltiorrhizaBurge (Danshen): A golden herbal medicine in cardiovascular therapeutics. Acta. Pharmacol. Sin..

[12] Chen W., Wang N., Li R.C., Xu G.F., Bao G., Jiang H.T., Wang M.D. (2018). Salvianolic acid B renders glioma cells more sensitive to radiation via Fis-1-mediated mitochondrial dysfunction. Biomed. Pharmacother..

[13] Chen Z., Zhang C., Gao F., Fu Q., Fu C., He Y., Zhang J. (2018). A systematic review on the rhizome of Ligusticum chuanxiong Hort. (Chuanxiong). Food Chem. Toxicol..

[14] Zuo H.L., Linghu K.G., Wang Y.L., Liu K.M., Gao Y., Yu H., Yang F.Q., Hu Y.J. (2020). Interactions of antithrombotic herbal medicines with Western cardiovascular drugs. Pharmacol. Res..

[15] Chang Y., Zhang D., Yang G., Zheng Y., Guo L. (2021). Screening of anti-lipase components of artemisia argyi leaves based on spectrum-effect relationships and HPLC-MS/MS. Front. Pharmacol..

[16] Wu P., Dong X., Song G.Q., Wei M.M., Fang C., Zheng F.B., Zhao Y.J., Lu H.Q., Cheng L.H., Zhou J.L. (2021). Bioactivity-guided discovery of quality control markers in rhizomes of Curcuma wenyujin based on spectrum-effect relationship against human lung cancer cells. Phytomedicine.

[17] Wang Y.L., Hu G., Zhang Q., Yang Y.X., Li Q.Q., Hu Y.J., Chen H., Yang F.Q. (2018). Screening and characterizing tyrosinase inhibitors from *Salvia miltiorrhiza* and *Carthamus tinctorius* by spectrum-effect relationship analysis and molecular docking. J. Anal. Methods Chem..

[18] Han J.H., Tan H., Duan Y.J., Chen Y.L., Zhu Y., Zhao B.C., Wang Y., Yang X.X. (2019). The cardioprotective properties and the involved mechanisms of NaoXinTong capsule. Pharmacol. Res..

[19] Wang Y.L., Zhang Q., Yin S.J., Cai L., Yang Y.X., Liu W., Hu Y.J., Chen H., Yang F.Q. (2019). Screening of blood-activating active components from Danshen-Honghua herbal pair by spectrum-effect relationship analysis. Phytomedicine.

[20] Zhang X.L., Liu L.F., Zhu L.Y., Bai Y.J., Mao Q., Li S.L., Chen S.L., Xu H.X. (2014). A high performance liquid chromatography fingerprinting and ultra high performance liquid chromatography coupled with quadrupole time-of-flight mass spectrometry chemical profiling approach to rapidly find characteristic chemical markers for quality evaluation of dispensing granules, a case study on Chuanxiong Rhizoma. J. Pharm. Biomed. Anal..

[21] Liang W.Y., Chen W.J., Wu L.F., Li S., Qi Q., Cui Y.P., Liang L.J., Ye T., Zhang L.Z. (2017). Quality Evaluation and chemical markers screening of Salvia miltiorrhiza Bge. (Danshen) based on HPLC fingerprints and HPLC-MS^n^ coupled with chemometrics. Molecules.

[22] Yang M., Liu A., Guan S., Sun J., Xu M., Guo D. (2006). Characterization of tanshinones in the roots of Salvia miltiorrhiza (Dan-shen) by high-performance liquid chromatography with electrospray ionization tandem mass spectrometry. Rapid Commun. Mass Spectrom..

